# Efficacy and mechanism of traditional Chinese medicine in relieving antibiotic-resistant bacterial diarrhea in children: study protocol for a randomized controlled trial

**DOI:** 10.1186/s13063-021-05381-8

**Published:** 2021-06-29

**Authors:** Chao-ran Bi, Wei Jing, Xiao-fei Xie, Yan-jing Liu

**Affiliations:** 1 College of Traditional Chinese Medicine, Changchun University of Chinese Medicine, 1035 Boshuo Road, Changchun, China; 2 Children’s Diagnosis and Treatment Center, Affiliated Hospital to ChangChun University of Chinese Medicine, 1478 Gongnong Road, Changchun, China; 3 Department of Endocrinology, Metabolism and Gastroenterology, Third Affiliated Clinical Hospital to Changchun University of Chinese Medicine, 1643 Jingyue Street, Changchun, China

**Keywords:** Bacterial diarrhea, Traditional Chinese medicine, Antibiotic resistance, Clinical efficacy evaluation, Mechanism study

## Abstract

**Background:**

Bacterial infection is an important cause of diarrhea in children, potentially leading to malnutrition, growth and development disorders, and even death. Antibiotic abuse and resistance are widespread problems worldwide, especially in China. We therefore designed a study to evaluate the clinical efficacy and mechanism of traditional Chinese medicine in alleviating the effects of antibiotic resistance in childhood bacterial diarrhea and enhancing the sensitivity of pathogenic bacteria to antibiotics.

**Methods:**

This randomized, double-blind, placebo-controlled clinical trial has completed ChiCTR registration. The trial will randomly divide 120 children who meet the inclusion criteria into three groups: experimental group 1 (basic treatment + Gegen Qinlian decoction granules + Erbai drink placebo), experimental group 2 (basic treatment + Erbai drink granules + Gegen Qinlian decoction placebo), and control group (basic treatment + Gegen Qinlian decoction placebo + Erbai drink placebo). The main efficacy indicators will be antibiotic use rate and clinical cure rate, and the secondary efficacy indicators will be time to antibiotic intervention, effective rate, and course of treatment determined after 5 days. The following physical and chemical indicators will be measured: routine blood parameters, procalcitonin, C-reactive protein, electrocardiogram, liver and kidney function, electrolytes, routine urinalysis, routine stool analysis, and stool culture (including drug sensitivity).

**Discussion:**

The results of this study may provide an objective clinical basis for the use of traditional Chinese medicine in managing antibiotic-resistant bacterial diarrhea in children, formulating relevant guidelines, and demonstrating the use of traditional Chinese medicine for reducing the use of antibiotics.

**Trial registration:**

Chinese Clinical Trial Registry ChiCTR1900027915. Last refreshed on December 4, 2019.

## Background

Antibiotic abuse and drug resistance are widespread throughout the world, especially in China. The overuse of antibiotics has particularly important implications for children. Infectious diarrhea is one of the most common diseases worldwide, potentially causing malnutrition, growth and development disorders, and is the most common disease and the leading cause of death in children under the age of 5 years in developing countries. Studies [1] have reported that the detection rate of *Shigella flexneri* in stool specimens of children with diarrhea in economically backward areas is 64.95%, suggesting that bacterial infection was an important cause of infectious diarrhea in children. There is thus a need to evaluate the clinical efficacy and mechanism of traditional Chinese medicine in alleviating the resistance of childhood bacterial diarrhea to antibiotics and enhancing the antibiotic sensitivity of pathogenic bacteria.

Bacterial diarrhea in children is a common pediatric disease caused by a variety of pathogens. The common clinical symptoms are increased stool frequency and changes in stool characteristics. The distribution of pathogenic bacteria in children with bacterial diarrhea shows regional characteristics. Wang et al. [2] found that patients with bacterial diarrhea in Lanzhou were mainly infected with pathogenic *Escherichia coli* and *Shigella*, and Wang et al. [3] found that *Shigella* was the main pathogen responsible for bacterial diarrhea in children under 14 years of age in Xi'an from 2004 to 2016, though the detection rate of *Salmonella* gradually increased over time. Yuan et al. [4] showed that the pathogenic bacteria in children with bacterial diarrhea in Taiyuan from 2002 to 2007 were mainly *E. coli*, with the incidence of *Shigella* decreasing year by year. Cui et al. [5] collected 10,881 strains of various diarrhea pathogens in 16 provinces and cities in China, including seven genera, 22 strains, and 90 serotypes, including 7632 strains of *Shigella* (70.14%), 1351 strains of *Vibrio* (12.42%), 981 strains of *Salmonella* (9.02%), 341 strains (3.13%) of diarrheal *E. coli*, 302 strains (2.78%) of *Aeromonas*, 269 strains (2.47%) of *Pseudomonas*, and five strains (0.05%) of *Yersinia enterocolitica*.

Antibiotics are the most commonly used treatment for drug-resistant bacterial diarrhea, and the rigorous, reasonable, and scientific use of antibiotics can guarantee the successful control and treatment of drug-resistant bacterial diarrhea. However the widening and inappropriate use, and even abuse, of antibiotics has exacerbated the problem of drug resistance. Hou et al. [6] found that *E. coli* had a higher rate of resistance to ampicillin (98.5%), compound trimethoprim (77.0%), and ceftriaxone (63.0%) compared with *Shigella*, based on drug sensitivity tests. Li and Liang [7] found that the susceptibility rates of various bacterial genera to antibiotics differed, with greater sensitivity (> 90%) to fourth-generation cephalosporins The order of drug resistance was penicillins, aminoglycosides, sulfonamides, penicillin compound preparations, quinolones, and cephalosporins, and no bacteria were resistant to carbapenems. Zhao et al. [8] suggested that pathogens were most sensitive to third-generation cephalosporins, followed by fluoroquinolones and aminoglycosides, and were resistant to other antibiotics.

Pediatric drug-resistant bacterial diarrhea belongs to the category of damp-heat syndrome of diarrhea in traditional Chinese medicine (TCM). TCM has always advocated “differentiation and treatment,” which has advantages for the treatment of drug-resistant bacterial diarrhea in children. Wang et al. [9] used Gegen Qinlian decoction to treat patients with acute infectious diarrhea (intestinal damp-heat syndrome), with good safety results. The effects of this agent may be related to reductions in of plasma levels of C-reactive protein (CRP), interleukin-6, tumor necrosis factor-α, and endotoxin levels.

Given that bacterial diarrhea in children is a common and serious problem mainly caused by *Shigella* and *Salmonella*, antibiotics are frequently used as the main treatment option. However, their misuse and abuse have led to increasing drug resistance among pathogenic bacteria. Although some laboratory studies [10, 11] found that Gegen Qinlian decoction had a significant inhibitory effect on *Shigella* and *Salmonella*, information based on clinical trials in children is lacking.

Erbai drink is an empirical prescription used by the master of traditional Chinese medicine, Wang Lie. This prescription is used to treat diarrhea of damp-heat type in children. However, in the existing guidelines [12], the prescription for pediatric damp-heat diarrhea is Gegen Qinlian Decoction, But, in clinical application, the master of Chinese medicine Wang Lie found that Erbai drink also has a significant effect in the treatment of diarrhea in children with damp-heat type. Wang et al. [13] summarize Wang Lie’s experience and found that Erbai drink is “harmonizing spleen yin and yang, clearing heat, promoting diuresis and detoxifying,” which is used to treat diarrhea in children with damp heat.

Erbai drink granules: Radix Paeoniae Alba (Bai Shao) 10 g, Rhizoma Atractylodis Macrocephalae (Bai Zhu)10 g, Radix scutellariae (Huang Qin) 10 g, Semen Coicis (Yi Yiren ) 10 g, Rhizoma Atractylodis (Cang Zhu) 5 g, Semen Plantaginis (Che Qianzi)10 g; Gegen Qinlian decoction granules: Radix Puerariae lobatae (Ge Gen) 10 g, Radix Scutellariae (Huang Qin) 10 g, Rhizoma Coptidis (Huang Lian) 5 g, Radix Glycyrrhizae (Gan Cao) 5 g. Two Chinese medicine prescriptions can all clear away heat, eliminate dampness ,and stop diarrhea for children with damp-heat diarrhea, but it can be found from the composition of the medicine that Gegen Qinlian decoction has a better heat-clearing effect and Erbai drink has a better dampness-eliminating effect. So, we hope that this difference can be verified in actual clinical observations.

Therefore, it is necessary to implement clinical trials to determine the safety and efficacy of TCM (Gegen Qinlian decoction and Erbai drink) in the treatment of antibiotic-resistant bacterial diarrhea in children.

## Methods/design

### Study aim

1. We aim to use Erbai drink and Gegen Qinlian decoction combined with antibiotics as intervention methods to evaluate the clinical efficacy of TCM for the treatment of bacterial diarrhea in children. We will also formulate clinical programs and guidelines for the treatment of pediatric bacterial diarrhea using TCM, to provide clinical evidence for the use of TCM in reducing the use of antibiotics.
2. Based on the evaluation of the clinical efficacy of the TCM preparations and their combined application, we aim to investigate the mechanism of TCM in alleviating antibiotic resistance and enhancing the sensitivity of pathogenic bacteria to antibiotics.
3. We aim to monitor the incidence of clinical adverse events/reactions, general physical examination items, and laboratory parameters.

### Study design

**Fig. 1:**
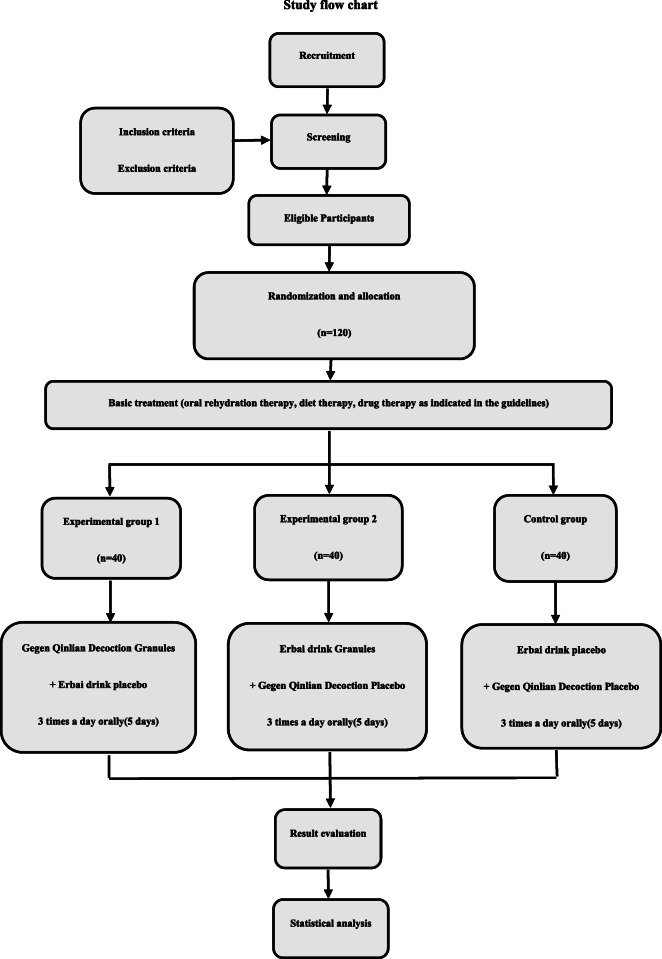
Study flow chart. Flow chart of enrolment, allocation, intervention, and assessment

This randomized, double-blind, placebo-controlled clinical trial will compare two different TCM preparations with a blank control group. The trial design is shown in Fig. 1.

### Setting and patients

A total of 120 children from the outpatient/inpatient departments of each participating center who meet the diagnostic criteria and inclusion criteria for bacterial diarrhea will be enrolled. The participating centers include the Children’s Diagnosis and Treatment Center of the Affiliated Hospital of Changchun University of Traditional Chinese Medicine, Shenzhen Children’s Hospital, and the First Affiliated Hospital of Hunan University of Traditional Chinese Medicine. The biological samples collected from the children must not be less than 2000. The children will be divided into three groups according to computer-generated random numbers, with a 1 in 3 probability of being allocated to any one group.

### Eligibility criteria

#### Diagnostic criteria

The Western medicine diagnostic criteria for drug-resistant bacterial diarrhea in children are as follows [14, 15]:

- Resistant bacteria found in the stool culture.
- Prerequisites: stool characteristics changed, showing loose, watery, mucus, or pus-blooded stools. Course of the disease within 2 weeks.
- Auxiliary conditions: stool frequency higher than usual, and at least three times a day, with or without abdominal pain, vomiting, and abdominal distension.

The TCM diagnostic criteria for diarrhea in children with damp-heat syndrome are as follows [16]:

- Main symptoms: watery stools, or like egg-drop soup, urgent diarrhea, frequent doses, and foul smell.
- Secondary symptoms: sometimes some mucus, abdominal pain, nausea and vomiting, or fever, irritability, thirst, yellow urine, red tongue, yellow and greasy coating, slippery pulse, purple hand venules.

#### Selection criteria

- Age from 6 months to 14 years.
- Meet the Chinese and Western medicine diagnostic criteria, course of the disease within 2 weeks, and condition mild to moderate.
- White blood cell count in routine stool ≥ 5/high-power field (HPF), or increased procalcitonin, percentage of CRP or neutrophils, or total number of white blood cells in routine blood.
- Able to obtain consent from a guardian, fill in the informed consent form, and agree to participate (children > 8 years old should sign a consent form).

#### Exclusion criteria

Patients meeting any of the following criteria will be excluded:

- Bacterial diarrhea caused by cholera, bacillary dysentery, typhoid fever, and paratyphoid fever (stool routine white blood cell count ≥ 15/HPF, and a few red blood cells).
- Children with severe dehydration, acidosis, shock, and immunodeficiency.
- Children with severe cardiovascular, liver, kidney, and hematopoietic diseases or with mental disorders.
- Acute diarrhea caused by intussusception and intestinal polyps.
- Diarrhea caused by surgery within 30 days before the clinical trial.
- Children who do not cooperate with treatment.
- Participated in other clinical trials within 1 month.
- Children with allergies to the known ingredients of the experimental drug.

#### Elimination criteria

Patients will be removed from the trial if they meet any of the following criteria:

- After selection, the patient is found not to meet the selection criteria or to meet the exclusion criteria.
- Patients who fail to follow the protocol after selection.
- Patients with no post-treatment visit records after selection.

#### Patient shedding criteria

- Subjects with poor compliance during the trial, affecting the safety and efficacy evaluation.
- Subjects with serious adverse events, complications, or specific physiological changes, who are not suitable to continue the trial.
- Children for whom trial blindness is broken.
- Children who choose to withdraw from the trial.
- When combined drugs, especially the drugs that have a greater impact on the effectiveness and safety of the experimental drug.
- Cases who withdraw from the trial, are lost to follow-up, or who die for before completing treatment.
- Cases with insufficient data, affecting the evaluation of effectiveness and safety.

#### Suspension criteria

- Severe adverse reactions during the medication.
- Major errors in the clinical trial plan making it difficult to evaluate the drug effect; or important deviations in implementation of the plan (e.g. blind bottom leakage, etc.), making it difficult to evaluate the drug effect.
- Decision to terminate the trial by the national regulatory agency, the Ministry of Science and Technology and other relevant project departments.

### Interventions

Both the experimental group and the control group will be treated with test drugs or placebo together with basic treatment.

#### Basic treatment

Oral rehydration treatment, dietary treatment, and drug treatment as specified in the guidelines.

1. Oral rehydration treatment: oral rehydration salt solution (Shanghai Johnson Pharmaceutical Co., Ltd.).
2. Diet treatment: carbohydrates and vegetables. Fasting high-protein, high-fat, and high-calorie foods.
3. Medication treatment: zinc supplementation (elemental zinc 20 mg/day for 10–14 days. Elemental zinc 20 mg is equivalent to 100 mg zinc sulfate and 140 mg zinc gluconate).

#### Test drugs

1. Experimental group 1: basic treatment + Gegen Qinlian decoction granules + Erbai drink placebo: Radix Puerariae lobatae (Ge Gen) 10 g, Radix Scutellariae (Huang Qin) 10 g, Rhizoma Coptidis (Huang Lian) 5 g, Radix Glycyrrhizae (Gan Cao) 5 g. (child’s dose, divided into three packets), orally in three times. Placebo (same taste and packaging as Erbai drink granules), children aged ≥ 6 months to ≤r1 year: 3/7 packs/time, three times a day orally; children aged > 1 year to ≤ 3 years: 1/2 pack/time, three times a day orally; children aged > 3 years to ≤ 5 years: 3/4 pack/time, three times a day orally; and children aged > 5 years to ≤ 14 years: 1 pack/time, three times a day orally.
2. Experimental group 2: basic treatment + Erbai drink granules + Gegen Qinlian decoction placebo: Radix Paeoniae Alba (Bai Shao) 10 g, Rhizoma Atractylodis Macrocephalae (Bai Zhu)10 g, Radix scutellariae (Huang Qin) 10 g, Semen Coicis (Yi Yiren ) 10 g, Rhizoma Atractylodis (Cang Zhu) 5 g, Semen Plantaginis (Che Qianzi)10 g (child’s dose, divided into three packs) orally in three times. Placebo (taste and packaging same as Gegen Qinlian decoction granules), children aged ≥ 6 months to ≤ 1 year: 3/7 packs/time, three times a day orally; children aged > 1 year to ≤ 3 years: 1/2 pack/time, three times a day orally; children aged > 3 years to ≤ 5 years: 3/4 pack/time, three times a day orally; and children aged > 5 years to ≤ 14 years: 1 pack/time, three times a day orally.
3. Control group: basic treatment + Erbai drink placebo + Gegen Qinlian decoction placebo: placebo (taste and packaging same as Erbai drink granules and Gegen Qinlian decoction granules), children aged ≥ 6 months to ≤ 1 year old: 3/7 packs/time, three times a day orally; children aged > 1 year to ≤ 3 years: 1/2 pack/time, three times a day orally; children aged > 3 years to ≤ 5 years: 3/4 pack/time, three times a day orally; and children aged > 5 years to ≤ 14 years: 1 pack/time, three times a day orally. Relevant antibiotics (ceftriaxone sodium for injection: 50 mg/kg/day, single intravenous infusion) can be added if required during the course of the illness, if there is an increase in the frequency of stools, a lack of improvement in stool characteristics, fever, increased proportion of neutrophils in the blood, increased CRP or procalcitonin, or increased routine white blood cell count in the stools.

One course of treatment for the above three groups will last 5 days, and one to two courses can be administered continuously. The specific observation time and related items are shown in Table 1.

**Table 1:** Specific observation time and related items

Stage		Enrollment	Clinical observation time (days)									
**Date of visit**		**0**	**1**	**2**	**3**	**4**	**5**	**6**	**7**	**8**	**9**	**10**
**Case screening**		**√**										
**Sign informed consent**		**√**										
**Random entry group**		**√**										
**Delivery of test drugs**		**√**										
**CRF completed**		**√**	**√**	**√**	**√**	**√**	**√**	**√◎**	**√◎**	**√◎**	**√◎**	**√◎**
**Basic medical history**		**√**										
**General information**		**√**										
**General inspection items**	**Physical examination**	**√**	**√**	**√**	**√**		**√**	**√◎**	**√◎**	**√◎**	**√◎**	**√◎**
	**Routine blood test**	**√**	**◎**	**◎**	**√**	**◎**	**◎**	**√◎**	**√◎**	**√◎**	**√◎**	**√◎**
	**Procalcitonin**	**√**	**◎**	**◎**	**√**	**◎**	**√**	**◎**	**◎**	**◎**	**◎**	**◎**
	**C-reactive protein**	**√**	**◎**	**◎**		**◎**	**◎**	**√◎**	**√◎**	**√◎**	**√◎**	**√◎**
	**Electrocardiogram**	**√**					**√**	**√◎**	**√◎**	**√◎**	**√◎**	**√◎**
	**Liver and kidney function**	**√**					**√**	**√◎**	**√◎**	**√◎**	**√◎**	**√◎**
	**Electrolytes**	**√**					**√**					
	**Routine urine test**	**√**					**√**					
	**Routine stools**	**√**	**◎**	**◎**	**√**	**◎**	**√**	**√◎**	**√◎**	**√◎**	**√◎**	**√◎**
	**Stool culture (including drug sensitivity)**	**√**					**√**	**√◎**	**√◎**	**√◎**	**√◎**	**√◎**
**Stool characteristics**		**√**	**√**	**√**	**√**	**√**	**√**	**√◎**	**√◎**	**√◎**	**√◎**	**√◎**
**Stool frequency**		**√**	**√**	**√**	**√**	**√**	**√**	**√◎**	**√◎**	**√◎**	**√◎**	**√◎**
**Symptom evaluation**		**√**	**√**	**√**	**√**	**√**	**√**	**√◎**	**√◎**	**√◎**	**√◎**	**√◎**
**Temperature card**		**√**	**√**	**√**	**√**	**√**	**√**	**√◎**	**√◎**	**√◎**	**√◎**	**√◎**
**Drug recovery**												
**Changes in drug use**			**√.**	**√.**	**√.**	**√.**	**√.**	**√.◎**	**√.◎**	**√.◎**	**√.◎**	**√.◎**
**Record complications**			**√.**	**√.**	**√.**	**√.**	**√.**	**√.◎**	**√.◎**	**√.◎**	**√.◎**	**√.◎**
**Combined medication**			**√.**	**√.**	**√.**	**√.**	**√.**	**√.◎**	**√.◎**	**√.◎**	**√.◎**	**√.◎**
**Adverse event**			**√.**	**√.**	**√.**	**√.**	**√.**	**√.◎**	**√.◎**	**√.◎**	**√.◎**	**√.◎**
**Causes of shedding**												

“√” means that it must be recorded; “√.” means that it should be recorded if it occurs; The part with this symbol “**◎**” means that you need to fill in if there is information; main symptoms and signs should be recorded until leaving the group; routine stools, stool culture, and safety indicators should be reviewed when leaving the group; efficacy determined on 5th day and patients who have not recovered should also be reviewed

### Outcome assessment

#### Primary outcome measures

Antibiotic use rate and clinical cure rate

#### Secondary outcome measures

Time to antibiotic intervention, effectiveness, and treatment course.

### Safety outcomes

Abnormal liver and kidney functions, electrocardiogram, and other results; allergic symptoms such as drug fever, allergic reactions, anaphylactic shock, or skin rash; severe vomiting, diarrhea, and other digestive system symptoms; cough, chest tightness, shortness of breath, and other respiratory symptoms; and all adverse events that occurred in the trial.

### Adverse events

Record the time, duration, extent, and treatment of adverse reactions. Evaluate its relevance to treatment.

### Data management

The computer software CDMS is used to establish the corresponding entry procedure and establish a special database system for this trial. Data entry adopts the double entry method, which is completed by two persons independently. Data review uses the verification function in computer software for logical inspection and automatic comparison. After the data is locked, the unit that saves the blind file submits the blind file to the data manager, and the data manager completes the unblinding of the data. The unblinded data is handed over to statistical analysts for analysis. Data that is significantly outside the clinically acceptable range must be verified, and the investigator must provide the necessary explanations.

After the completion of the trial, the informed consent form and the subject’s signature code form will be kept by each hospital. The clinical trial data will be kept until 5 years after the termination of the clinical trial.

### Statistical analysis

Statistical analysis will be carried out using SAS 9.4 or above. According to the data characteristics, measured data will be analyzed by independent sample *t* tests or rank sum tests for comparison between groups and numerical data will be analyzed by χ^2^or Fisher’s exact probability tests. Laboratory data for all clinical endpoints will be analyzed according to intention to treat (ITT) and compliance (PP) analyses, and safety analysis will be performed using a safety data set (SS). The efficacy scores at the time of patient enrollment, intermediate follow-up (day 3, day 4 to day 7 after treatment), and at the end point of the trial will be calculated, and the mean, standard deviation, median, P5, P75, maximum and minimum values of the differences in values between baseline and the treatment period will be used. PP and ITT will be adopted to analyze the main index and overall index. Subjects with positive urine culture indicators will be included in the statistical subgroup to analyze the correlation between the study program and the overall efficacy evaluation index. Because this is a multicenter, randomized, double-blind controlled trial, the possible central effect on efficacy indicators should be considered.

### Quality control

#### Sample size

We calculated the sample size according to our primary study. We found that the use rate of antibiotics in the two groups of patients treated with traditional Chinese medicine was 46% and that of the control group was 80%. Calculate the sample size under the settings of α = 0.05 and β = 0.80. The grouping ratio is 1:1:1. Calculate the single group n = 37, so there are 111 cases in the three groups. Taking into account a drop-out of 10%, In the end, we set the number of cases to 120.

#### Randomization and allocation

Taking the center as the unit, the patients were divided into an experimental group and a control group according to the stratified randomization principle. (Through the “central code allocation random number” sequence of SAS statistical software, the random code of each center is obtained, and the research center is randomly assigned through the random code.)

#### Blinding

Personnel were blinded to the scale evaluation, data collection, laboratory index testing, and statistics.

#### Drug control

Design a patient diary to collect information on study medication management, symptom assessment, and adverse events.

#### Auditing

An independent inspection center was established, and the inspection center appointed inspectors to conduct systematic inspections of trial-related activities and documents.

#### Confidentiality

All information about this trial must be kept strictly confidential, and documents that can show the identity of the subject are kept strictly confidential.

## Discussion

The high incidence of bacterial diarrhea in children and the widespread use of antibiotics in clinical practice mean that drug resistance has become an increasingly serious problem. This study aims to evaluate the clinical efficacy of TCM for treating pediatric bacterial diarrhea by carrying out a clinical trial of Gegen Qinlian Decoction and Erbai drink in children with bacterial diarrhea. The results will hopefully allow the formulation of relevant clinical programs and corresponding guidelines. The results may also provide clinical evidence to support the use of TCM for reducing the use of antibiotics.

## Trial status

Protocol version V1.0 dated 20 March 2019. Trial patients were being recruited at the time of this submission. The first participant was enrolled on April 1, 2020. Recruitment will be complete on 31 December 2021.

## Data Availability

All data collected during the trial will be available from the corresponding author (Yan-jing Liu,2785118375@qq.com) for anyone who wishes to access the data immediately following publication.

## Abbreviations

TCM: Traditional Chinese medicine
CRP: C-reactive protein
HPF: High-power field
